# Implementation of genome-wide complex trait analysis to quantify the heritability in multiple myeloma

**DOI:** 10.1038/srep12473

**Published:** 2015-07-24

**Authors:** Jonathan S. Mitchell, David C. Johnson, Kevin Litchfield, Peter Broderick, Niels Weinhold, Faith E. Davies, Walter A. Gregory, Graham H. Jackson, Martin Kaiser, Gareth J. Morgan, Richard S. Houlston

**Affiliations:** 1 Molecular and Population Genetics, Division of Genetics and Epidemiology, The Institute of Cancer Research, Surrey, UK; 2 Department of Haemato-Oncology, Division of Pathology, The Institute of Cancer Research, Surrey, UK; 3 Myeloma Institute for Research and Therapy, University of Arkansas for Medical Sciences, Little Rock, AR, USA; 4 Leeds Institute of Molecular Medicine, Section of Clinical Trials Research, University of Leeds, Leeds, UK; 5 Royal Victoria Infirmary, Newcastle upon Tyne, UK; 6 Centre for Myeloma Research, Division of Molecular Pathology, The Institute of Cancer Research, Sutton, UK

## Abstract

A sizeable fraction of multiple myeloma (MM) is expected to be explained by heritable factors. Genome-wide association studies (GWAS) have successfully identified a number of common single-nucleotide polymorphisms (SNPs) influencing MM risk. While these SNPs only explain a small proportion of the genetic risk it is unclear how much is left to be detected by other, yet to be identified, common SNPs. Therefore, we applied Genome-Wide Complex Trait Analysis (GCTA) to 2,282 cases and 5,197 controls individuals to estimate the heritability of MM. We estimated that the heritability explained by known common MM risk SNPs identified in GWAS was 2.9% (±2.4%), whereas the heritability explained by all common SNPs was 15.2% (±2.8%). Comparing the heritability explained by the common variants with that from family studies, a fraction of the heritability may be explained by other genetic variants, such as rare variants. In summary, our results suggest that known MM SNPs only explain a small proportion of the heritability and more common SNPs remain to be identified.

Multiple myeloma (MM) is a malignancy of the plasma cells^1^. In the United Kingdom, approximately 4,800 individuals are diagnosed each year with MM and approximately 2,700 die from the cancer (http://www.cancerresearchuk.org/cancer-info/cancerstats/types/myeloma/uk-multiple-myeloma-statistics). Monoclonal gammopathy of undetermined significance (MGUS; a pre-malignant clone of plasma cells producing a monoclonal paraprotein) is present in ~2% of individuals older than 50 years, and the risk of progressing to MM increases by 1% each year^2^. The increased risk of MM in the relatives of individuals with MGUS is consistent with MGUS being a marker of genetic susceptibility^3^,^4^. To date, no lifestyle or environmental exposure factors have been consistently linked to an increased risk of MM or MGUS^4^.

Recent genome-wide association studies (GWAS) have provided the first unambiguous evidence for genetic susceptibility to MM identifying single nucleotide polymorphisms (SNPs) affecting risk at chromosomes 2p33.3, 3p22.1, 3q26.2, 6p21.33, 7p15.3, 11q13, 17p11.2, and 22q13.1^5^,^6^,^7^. It is not however apparent how much of the heritability of MM these SNPs collectively explain. Moreover as the SNPs identified by GWAS have to pass a very stringent significance threshold, there are likely to be multiple SNPs with weak effect sizes that do not pass the threshold but still contribute to the heritability. Quantifying the heritability explained by both known and potential susceptibility SNPs is important in explaining the aetiological basis of MM and understanding its genetic architecture.

Genome-wide Complex Trait Analysis (GCTA) estimates the polygenic variance (*i.e.* heritability) ascribable to all GWAS SNPs simultaneously irrespective of whether they pass a certain significance threshold^8^,^9^,^10^. GCTA calculates the genetic similarity between subjects and uses the restricted maximum-likelihood approach to estimate narrow sense heritability. An alternative approach based on phenotype correlation-genotype correlation (PCGC) regression has been developed to avoid the bias introduced by GCTA when applied to case-control studies^11^. It has been proposed that for disease traits GCTA introduces an error when making the necessary conversion from the heritability calculated on the observed binary disease phenotype to the unobserved liability scale. In this article we have explored the MM heritability explained by common SNPs using both GCTA and PCGC approaches.

## Results

As previously advocated when calculating the heritability of a disease such as a cancer^12^,^13^ we used lifetime risk rather than prevalence to transform data to the liability scale. After applying this procedure to account for the lifetime risk of MM (0.00739 ± 0.00014) and ascertainment the heritability of MM which can be explained by considering all SNPs using GCTA was estimated to be 15.2% (±2.8%). Heritability was found not to be sensitive to our assumed lifetime risk within 1 standard error (15.1% to 15.2%). The estimated heritability from PCGC regression was 16.8% (±4.1%). Due to the similarity of the two results we focus further analyses and discussion solely on the GCTA results, but report the PCGC estimates for completeness. To adjust for the underestimate of heritability caused by the array SNPs not being in complete LD with the causal SNPs we follow the procedure of Yang *et al.*^14^. The MAF distribution of causal SNPs affects this estimate, and as we do not know the true distribution we calculate the adjustment for a range of MAF thresholds (Table 1). Assuming that causal SNPs and array SNPs have the same distribution (MAF threshold of 0.5) the adjusted heritability was calculated to be 17.3% (±3.2%) which is close to the unadjusted value of 15.2% (±2.8%). Conversely if causal SNPs are assumed to have MAF < 0.1 then the adjusted heritability is 27.8% (±5.1%) which is significantly higher than the unadjusted value. While it is expected from neutral and selection theories of quantitative genetic variation that causal SNPs will on average have lower MAF than those on the array^15^ the exact distribution of MAF for MM causal SNPs is unknown.

**Figure 1:**
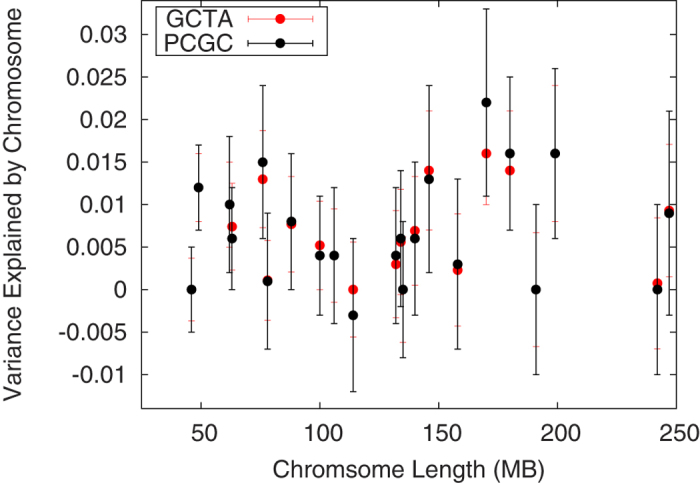
Variance explained by each chromosome as a function of chromosome length.

To gain insight into the underlying basis of the heritability associated with common variation we investigated the relative contribution of individual chromosomes (Supplementary Table 1). In contrast to a trait such as height where there is a strong linear relationship between chromosome length and the variance explained by the chromosome^10^ we found no such relationship (R^2^ = 0.0063 from GCTA and R^2^ = 0.0016 from PCGC analysis) (Fig. 1).

To investigate further the distribution of heritability along the genome we estimate the heritability associated with the seven risk loci previously discovered from GWAS by including the risk SNP genotype as a covariate (Table 2). The total heritability of MM which is explained by the seven risk loci is 2.9% (±2.4%). This is in good agreement with the value of 2.5% (±0.4%) obtained by calculating the heritability associated with the genetic relationship matrix (GRM) of risk loci. These estimates are substantially lower (by 12.3% and 12.7%) than the genetic variance associated with all the SNPs on the array. These data therefore suggest that a large proportion of the heritability in MM remains unaccounted for by current GWAS.

To quantify the importance of transcript regions to MM heritability, we partitioned the variance explained by all the SNPs onto transcript and non-transcript regions of the genome. The Variant Effect Predictor program was used to determine which SNPs were on transcript regions. We calculated the heritability due to transcript regions to be 9.7% (±2.4%) and non-transcript regions to be 5.5% (±2.1%). Transcript regions were shown to explain more variation than non-transcript regions despite covering a far smaller fraction of the genome. However, the error introduced from incomplete LD is a particular problem in this analysis because the majority of array SNPs map to transcripts (67%).

Multiple myeloma can be broadly classified into hyperdiploid and non-hyperdiploid subtypes, with further subdivision based on the presence of *IGH*@ translocations, the most common of which are t(11;14)(q13;q32) and t(4;14)(p16;q32)^16^. These translocations contribute directly to the development of the different MM subtypes. The observation that the *CCND1* c.870G > A polymorphism is a risk factor for t(11;14)(q13;q32) MM^7^ supports the hypothesis that the different MM subtypes are likely to have different aetiologic pathways. To explore the possibility that heritability basis of MM might be subtype dependent we conducted a stratified analysis. While heritability estimates showed differences these were not statistically significant. Respective estimates for hyperdiploid, translocation MM, t(11;14) and t(4;14) MM being: 0.143 ± 0.069, 0.077 ± 0.081, 0.068 ± 0.196 and 0.236 ± 0.250.

## Discussion

In this study, we found that the MM susceptibility SNPs thus far identified by GWAS explain only a small proportion of the MM heritability. In contrast, the explained heritability was significantly greater when considering the combined effect of all common SNPs. Our estimates of phenotypic variance therefore provide unequivocal evidence of yet-to-be-discovered genetic variants contributing to the development of MM. The estimates are based on realized relationships between very distant relationships thereby breaking up possible correlation (*i.e*. confounding) between genetic and environmental risk factors. The liability threshold model upon which heritability is estimated assumes the distribution of MM liability is unimodal. Since no major gene for MM has been identified or no lifestyle or environmental factor robustly implicated our estimate of heritability is unlikely to be biased.

In GCTA, a prevalence parameter is required in order to transform the estimated heritability from the observed scale to liability scale. As has been previously advocated when calculating the heritability of disease we use the lifetime risk^13^. Previous work has however demonstrated that prevalence values have only a small impact on GCTA heritability estimates; for example Do *et al.*^17^ showed that 3-fold difference in assumed prevalence (0.005–0.015) affected estimates of heritability by only 6.7%.

Large-scale cohort analysis has shown that the risk of MM is increased 2.45-fold in relatives of MM patients^3^. Our estimated heritability explained by all common SNPs is 15.2%, which translates to common variation accounting for approximately 62% of the familial risk associated with MM. This is likely however to be an underestimate since our estimate of heritability provides a lower bound for narrow-sense heritability, due to imperfect LD between genotyped SNPs and causal variants. Furthermore, indel and structural variants were not considered, although some may be tagged. In addition, the portion of variance explained by GWAS SNPs is underestimated by GCTA, since the model imposes a prior centered zero as the effect size of the SNPs used in calculation of the GRM.

It is possible that some disease-causing variants which are very rare have a substantive effect on MM risk but there is no reason to believe that all of the unexplained genetic variance is solely explained by a restricted number of high-risk mutations. Analysis of additional ongoing GWAS of MM, which are based on higher density array technology, are therefore likely to be informative in refining estimates of heritability. Moreover, higher-density SNP genotyping would provide a higher probability of LD with functional disease-causing variants thus potentially affording the capturing of a higher proportion of the genetic variance—provided the characteristics of disease-causing variants do not differ systematically from the genotyped SNPs (*e.g.* because of lower MAF).

Here we have assumed that the genetic susceptibility to MM is defined by alleles inherited in a Mendelian fashion. It is entirely possible *de novo* copy number variants and/or methylation status variants also contribute to MM risk. Furthermore, the heritability estimated in our analysis is simply the additive variance as a proportion of the phenotypic variance. Thus, it does not include non-additive genetic variance (gene-gene interactions or dominance effects) or gene-environment interactions impacting on MM risk. Additionally, it has recently been proposed that epistatic gene-gene interactions may play a significant role in mediating the development of complex traits and underscore “phantom heritability”, that is, the apparent missing heritability from purely additive genetic effects. It is therefore possible that our estimates of heritability are inherently conservative in terms of defining the contribution of the impact of inherited predisposition to MM. Notwithstanding such caveats the magnitude of the estimated heritability in our study is such that this polygenic susceptibility contributes significantly to the development of MM.

In summary, we report the first study to show that a large proportion of the heritability of developing MM can be ascribed to common genetic variation. Moreover, it is the first to show biologically and unequivocally that the risk of MM is highly polygenic. Not only do our findings provide quantification of the impact of common variation on MM risk, they also provide a strong rationale for continuing to search for additional novel risk variants through GWAS-based strategies. It is, therefore, likely that additional novel risk variants will have more modest effect on MM risk than those which have been so far discovered. Full mapping of all common SNPs associated with MM may plausibly offer utility in personalized risk profiling for the disease, through construction of polygenic risk scoring (PRS) models, as implemented in other cancer types^18^,^19^,^20^. A preliminary PRS model^21^ for MM assuming a log-normal relative risk distribution with a fully mapped set of common MM risk SNPs would mean individuals in the top 1% of the population distribution of risk would have a 6.29-fold increased risk, equating to a 5% lifetime risk of MM. In a future context where population level genomic testing may become routine practice, individuals at significantly elevated risk of MM would be identifiable through inspection of their common SNP risk profile.

Future analyses incorporating denser genome and exome-wide assays in conjunction with newer sequencing technologies will likely see increased heritability estimates associated with MM and other complex traits, as a significantly larger genetic contribution to disease risk is identified. Such analyses are going to benefit greatly from the development of large consortia such as MAGIC^22^.

## Materials and Methods

### Subjects

The study is based on an expanded previously published GWAS of MM^6^. In brief, 1,371 MM cases were recruited through the UK Medical Research Council (MRC) Myeloma-IX trial^23^ and 1,008 MM cases through Myeloma-XI (http://ctru.leeds.ac.uk/myelomaXI). The Myeloma-IX trial was approved by the MRC Leukaemia Data Monitoring and Ethics committee (MREC 02/8/95, ISRCTN68454111) and the Myeloma-XI trial was approved by the Oxfordshire Research Ethics Committee (MREC 17/09/09, ISRCTN49407852).

### Quality control

Detailed information and quality control procedures have been previously described in detail^6^. In brief, genotyping of cases was conducted using Illumina OmniExpress BeadChips. Genotype frequencies were compared with publicly accessible genotype data generated by the UK Wellcome Trust Case Control Consortium 2 (WTCCC2) study of 2,699 individuals from the 1958 British Birth Cohort (known as 58C)^24^ and 2,501 individuals from the UK Blood Service (UKBS) collections that had been genotyped using Illumina Human 1.2M-Duo Custom_v1 Array BeadChips.

Genotype data were filtered on the basis of pre-specified quality control measures. Individual SNPs were excluded if they showed deviation from Hardy-Weinberg equilibrium with *P* < 1.0 × 10^−6^ in controls, an individual SNP genotype yield <95% or a minor allele frequency <1%. After this filtering, 408,422 SNPs common to both case-control series remained for further study. A total of 97 case samples were removed during quality control processing for several reasons, including if sample genotyping failed (call rate < 95%), if samples belonged to pairs of unknown duplicates or closely related individuals (IBS > 0.80) or if individuals were of different ancestry from the cohort of Utah residents of Northern and Western European descent (CEU) (Supplementary Figure 1). A quantile-quantile (Q-Q) plot of genome-wide association test statistics showed minimal inflation, rendering substantial cryptic population substructure unlikely (genomic inflation factor^25^, *λ* = 1.028; Supplementary Figure 2). After QC the study comprised 2,282 cases and 5,197 controls.

### Statistical analysis

We used GCTA to estimate MM heritability under a number of different scenarios; heritability being defined as the proportion of phenotypic variation in a population that is due to genetic variance between individuals. A genetic relationship matrix (GRM) of pairs of samples was used as input for the restricted maximum likelihood analysis to estimate the heritability explained by the selected set of SNPs. GCTA uses the disease prevalence to transform the estimated heritability to the liability scale. As previously advocated when calculating the heritability of a disease such as a cancer^12^,^13^ we used the lifetime risk which for MM is estimated to be 0.00739 ± 0.00014 (http://www.cancerresearchuk.org/cancer-info/cancerstats/types/myeloma/incidence/uk-multiple-myeloma-incidence-statistics#Lifetime). The analyses were not adjusted for eigenvectors from principal component analysis as the inflation factor was found to be close to 1.

We estimated the heritability under the following scenarios:

1. Heritability explained by the autosome. A single GRM is computed for all autosomal SNPs.
2. Heritability explained by individual chromosome. A GRM is computed for each chromosome individually and then fitting is done simultaneously for all chromosome GRMs using the REML approach.
3. Heritability explained by risk SNPs identified by GWASs as located within autosomal regions associated with MM. For each risk SNP the heritability is estimated for all chromosomes simultaneously using the risk SNP genotype as a covariate. The heritability associated with the SNP is taken to be the difference between the heritability of the chromosome on which it is found as calculated with and without covariate. As an alternative test a GRM is computed for all SNPs within 500 kbp of the seven identified risk SNP. The heritability associated with all these SNPs is calculated by fitting simultaneously this GRM and a GRM containing all autosomal SNPs excluding these.
4. Heritability explained by transcript and non-transcript regions. A separate GRM is computed for SNPs in transcript regions and those not on transcript regions as defined by the Variants Effect Predictor (VEP)^26^. Heritability is estimated simultaneously for the two GRM using the REML approach.

This analysis was repeated with PCGC using the same GRM as input to estimate the heritability using regression.

## Additional Information

**How to cite this article**: Mitchell, J. S. *et al.* Implementation of genome-wide complex trait analysis to quantify the heritability in multiple myeloma. *Sci. Rep.* **5**, 12473; doi: 10.1038/srep12473 (2015).

## Supplementary Material

Supplementary Information

## Figures and Tables

**Table 1:** Heritability of multiple myeloma adjusted for incomplete LD between causal SNPs and those used to compute the GRM.

MAF Threshold	Heritability	
	GCTA	PCGC
No adjustment	0.152 ± 0.028	0.168 ± 0.041
0.5	0.173 ± 0.032	0.192 ± 0.049
0.4	0.180 ± 0.033	0.200 ± 0.049
0.3	0.192 ± 0.035	0.212 ± 0.058
0.2	0.212 ± 0.039	0.235 ± 0.070
0.1	0.278 ± 0.051	0.307 ± 0.079

Various MAF thresholds were used to simulate different possible MAF distributions of the causal SNPs.

**Table 2:** Estimates of the variance explained by individual multiple myeloma risk SNPs.

Risk SNP	Position	Fraction of Variance Explained	
		GCTA	PCGC
rs6746082	2p23.3	0.00074 ± 0.011	0.005 ± 0.016
rs1052501	3p22.1	0.005 ± 0.010	0.005 ± 0.014
rs10936599	3q26.2	0.0074 ± 0.010	0.007 ± 0.014
rs2285803	6p21.3	0.0056 ± 0.0089	0.013 ± 0.013
rs4487645	7p15.3	0.0023 ± 0.0093	0.006 ± 0.013
rs4273077	17p11.2	0.000068 ± 0.0067	0.000 ± 0.011
rs877529	22q13.1	0.0078 ± 0.0059	0.007 ± 0.007
Total		0.029 ± 0.024	0.043 ± 0.034
