# Supplementary material for: Implementation of genome-wide complex trait analysis to quantify the heritability in multiple myeloma
Source: Sci Rep. 2015 Jul 24;5:12473. doi: 10.1038/srep12473 (PMC4513545; doi:10.1038/srep12473)
Supplement: Supplementary Information [file srep12473-s1.doc]

**­­­SUPPLEMENTARY INFORMATION**

**Implementation of genome-wide complex trait analysis to quantify the heritability in multiple myeloma**

Jonathan S. Mitchell, David C. Johnson, Kevin Litchfield, Peter [Broderick](http://www.ncbi.nlm.nih.gov/pubmed?term=Broderick P%5BAuthor%5D&cauthor=true&cauthor_uid=23955597), Niels Weinhold, Faith E. [Davies](http://www.ncbi.nlm.nih.gov/pubmed?term=Davies FE%5BAuthor%5D&cauthor=true&cauthor_uid=23955597), Walter A. [Gregory](http://www.ncbi.nlm.nih.gov/pubmed?term=Gregory WA%5BAuthor%5D&cauthor=true&cauthor_uid=23955597), Graham H. [Jackson](http://www.ncbi.nlm.nih.gov/pubmed?term=Jackson GH%5BAuthor%5D&cauthor=true&cauthor_uid=23955597), Martin Kaiser, Gareth J. [Morgan](http://www.ncbi.nlm.nih.gov/pubmed?term=Morgan GJ%5BAuthor%5D&cauthor=true&cauthor_uid=23955597), Richard S. Houlston

**Supplementary Figure 1**: **Identification of individuals of non-European ancestry in cases and controls.** The first two principal components of the analysis were plotted. HapMap CEU individuals are plotted in blue; CHB individuals are plotted in indigo; JPN are plotted in cyan; YRI individuals are plotted in yellow. Cases are plotted in red and have been removed if existing outside of the Controls (green).


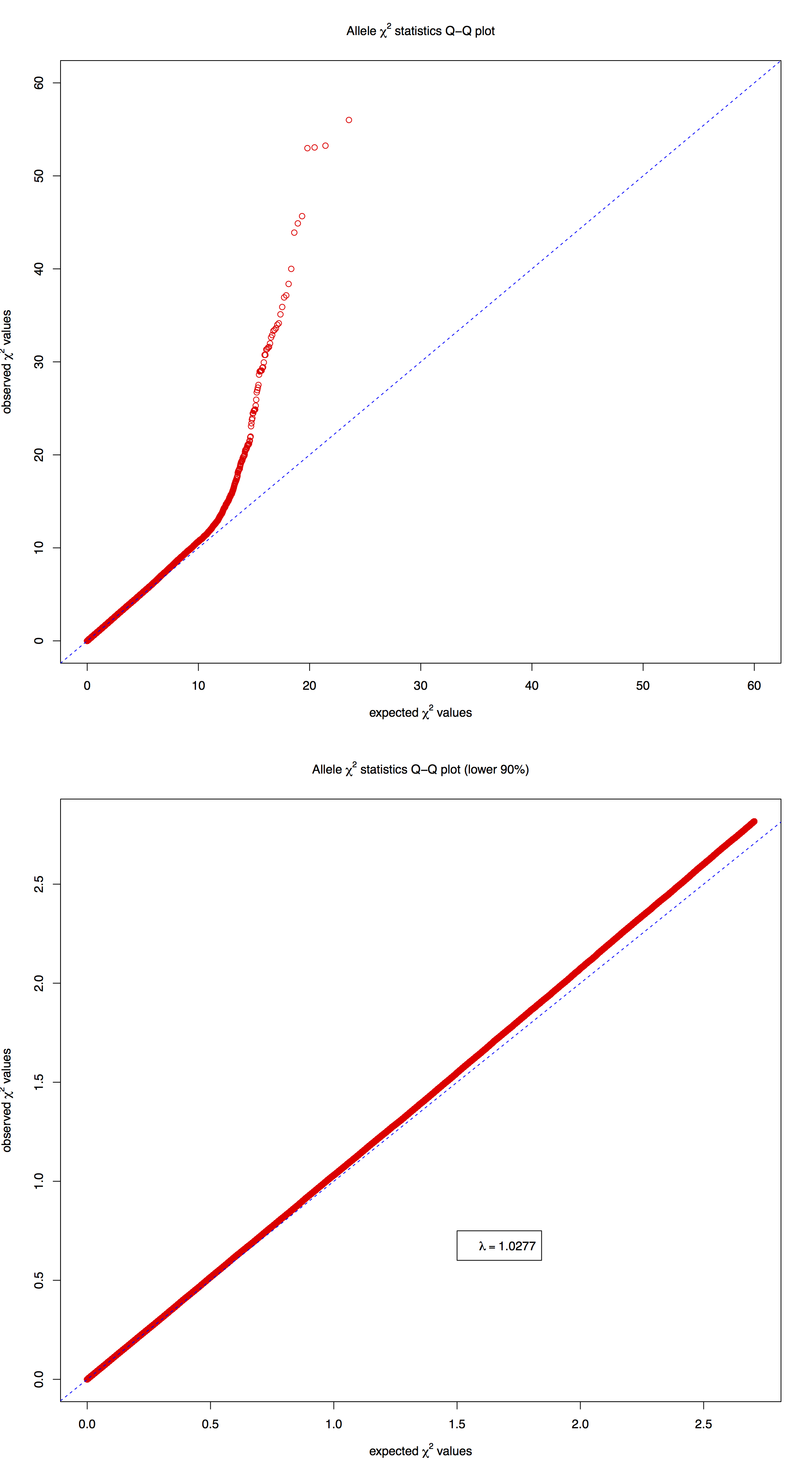


**Supplementary Figure 2:** **Q-Q plots of observed *P*-values (-log_10_*P*) for association.** The blue line represents the null hypothesis of no true association.

| Chromosome | Fraction of Variance Explained | |
| --- | --- | --- |
|  | GCTA | PCGC |
| 1 | 0.0093 ± 0.0078 | 0.009 ± 0.012 |
| 2 | 0.00074 ± 0.0077 | 0.00 ± 0.01 |
| 3 | 0.016 ± 0.008 | 0.016 ± 0.01 |
| 4 | 0.0000 ± 0.0067 | 0.00 ± 0.01 |
| 5 | 0.014 ± 0.007 | 0.016 ± 0.009 |
| 6 | 0.016 ± 0.006 | 0.022 ± 0.011 |
| 7 | 0.0023 ± 0.0066 | 0.003 ± 0.010 |
| 8 | 0.014 ± 0.007 | 0.013 ± 0.011 |
| 9 | 0.0069 ± 0.0064 | 0.006 ± 0.009 |
| 10 | 0.0000 ± 0.0062 | 0.000 ± 0.008 |
| 11 | 0.0056 ± 0.0062 | 0.006 ± 0.008 |
| 12 | 0.0030 ± 0.0063 | 0.004 ± 0.008 |
| 13 | 0.0000 ± 0.0056 | -0.003 ± 0.009 |
| 14 | 0.0040 ± 0.0055 | 0.004 ± 0.008 |
| 15 | 0.0052 ± 0.0052 | 0.004 ± 0.007 |
| 16 | 0.0077 ± 0.0056 | 0.008 ± 0.008 |
| 17 | 0.0011 ± 0.0047 | 0.001 ± 0.008 |
| 18 | 0.013 ± 0.0057 | 0.015 ± 0.009 |
| 19 | 0.0074 ± 0.0051 | 0.006 ± 0.006 |
| 20 | 0.010 ± 0.005 | 0.010 ± 0.008 |
| 21 | 0.0000 ± 0.0037 | 0.000 ± 0.005 |
| 22 | 0.012 ± 0.004 | 0.012 ± 0.005 |
| Total | 0.15 ± 0.03 | 0.15 ± 0.04 |

Supplementary Table 1: Estimates of the variance explained by individual chromosomes.
